# Equilibrium Fractionation of Oxygen Isotopes in the U_3_O_8_–Atmospheric Oxygen System

**DOI:** 10.1021/acsomega.2c06148

**Published:** 2022-12-05

**Authors:** Maor Assulin, Ruth Yam, Anna Kossoy, Eyal Elish, Aldo Shemesh

**Affiliations:** † Department of Earth and Planetary Sciences, Weizmann Institute of Science, Rehovot76100, Israel; ‡ Analytical Chemistry Department, Nuclear Research Center Negev (NRCN), Beer Sheva84190, Israel; § Department of Chemical Research Support, Weizmann Institute of Science, Rehovot76100, Israel

## Abstract

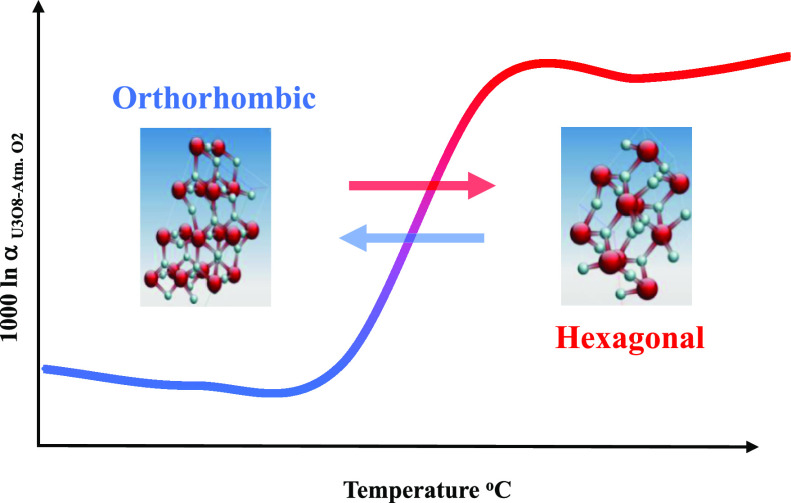

As a major component in the nuclear fuel cycle, octoxide uranium is subjected to intensive nuclear forensics research. Scientific efforts have been mainly dedicated to determine signatures, allowing for clear and distinct attribution. The oxygen isotopic composition of octoxide uranium, acquired during the fabrication process of the nuclear fuel, might serve as a signature. Hence, understanding the factors governing the final oxygen isotopic composition and the chemical systems in which U_3_O_8_ was produced may develop a new fingerprint concerning the history of the material and/or the process to which it was subjected. This research determines the fractionation of oxygen isotopes at different temperatures relevant to the nuclear fuel cycle in the system of U_3_O_8_ and atmospheric O_2_. We avoid the retrograde isotope effect at the cooling stage at the end of the fabrication process of U_3_O_8_. The system attains the isotope equilibrium at temperatures higher than 300 °C. The average δ^18^O values of U_3_O_8_ in equilibrium with atmospheric oxygen have been found to span over a wide range, from −9.90‰ at 300 °C up to 18.40‰ at 800 °C. The temperature dependency of the equilibrium fractionation (1000 ln α_U_3_O_8_-atm. O_2__) exhibits two distinct regions, around −33‰ between 300 °C and −500 °C and −5‰ between 700 °C and −800 °C. The sharp change coincides with the transition from a pseudo-hexagonal structure to a hexagonal structure. A depletion trend in δ^18^O is associated with the orthorhombic structure and may result from the uranium mass effect, which might also play a role in the depletion of 5‰ versus atmospheric oxygen at high temperatures.

## 1 Introduction

The fractionation of oxygen isotopes (^16^O, ^17^O, and ^18^O) among phases is an important measure applied in many scientific fields, such as geochemistry,^1^ climate reconstruction,^2,3^ and high-temperature magmatic and metamorphic processes,^4−6^ to trace chemical reactions and environmental conditions. In the last 2 decades, several studies have investigated the use of oxygen isotopic composition in various uranium oxides as a signature for nuclear forensics applications.^7−17^ Triuranium octoxide (U_3_O_8_), the most stable form of uranium oxide, is a key compound in the uranium nuclear fuel cycle. U_3_O_8_ is an intermediate product in the front end of the production cycle of the uranium nuclear fuel and a major compound in nuclear waste due to its thermal and chemical stabilities.^18−21^ Understanding the mechanism governing the final oxygen isotopic composition of U_3_O_8_ is fundamental to deciphering the material history in view of nuclear forensics investigations.

The production processes of U_3_O_8_ in the nuclear fuel cycle differ among the various producers in starting materials and fabrication temperatures, mainly in the range of 500–800 °C.^21^ However, U_3_O_8_ is manufactured under an atmospheric environment with full exposure to atmospheric oxygen.

A series of studies investigated the main parameters of the manufacturing process, which determine the final oxygen isotopic composition (δ^18^O) of U_3_O_8_. The effects of calcination temperature, calcination time, different starting materials, different initial solutions, air humidity, and the cooling rate of the products were studied.^7,11,12,15−17,22^ Plaue^11^ synthesized U_3_O_8_ from the U metal and different UO_2_ samples and showed increased δ^18^O values with increasing temperature. Klosterman et al.^16^ synthesized U_3_O_8_ from uranium peroxide at 300–1000 °C and reported a fractionation of about −22‰ up to about −5‰, respectively, between the oxide and atmospheric oxygen with a retrograde isotope effect. Klosterman et al.^22^ determined the fractionation of oxygen isotopes between U_3_O_8_ and N_2_/O_2_ atmosphere at 400, 600, and 800 °C, and different calcination times (between 1 and 72 h) reported an average of Δ^18^O UO_*x*_-O_2_ of −55.1‰ ± 1.0‰, −17.4‰ ± 2.2‰, and −8.2‰ ± 0.7‰, respectively. Assulin et al.^17^ synthesized U_3_O_8_ in temperatures ranging from 650 to 850 °C and calcination times ranging from 30 min up to 168 h, concluding that under atmospheric conditions, the δ^18^O of U_3_O_8_ is independent of the starting materials and of the original oxygen isotopic composition of UO_3_ from which it had been prepared. In addition, the δ^18^O values of synthetic U_3_O_8_ were found to be similar and did not change as a function of calcination time or calcination temperature. Assulin et al.^17^ showed that the cooling profile at the end of the fabrication process of U_3_O_8_ determines the final oxygen isotopic composition, yielding a significant isotope effect in the order of 30‰, and that the interaction with atmospheric oxygen is the primary process that controls the δ^18^O value of U_3_O_8_. The common conclusion of the above studies is that the reaction with atmospheric oxygen is a crucial factor in determining the final oxygen isotopic composition of U_3_O_8_.

Exchange experiments provide a means to directly determine the fractionation factors or equilibrium constants. Early studies of kinetic and equilibrium isotope-exchange reactions between solid and gas^23^ classified the exchange between U_3_O_8_ and UO_2_ and oxygen gas as the heterophase exchange but did not specify the mechanism through which the exchange occurs.

The isotopic exchange reaction involves changes in the vibrational frequencies of the lattice. The crystal structure influences the oxygen isotopic properties depending on the differences between the U–O bond energy and the interstitial sites occupied by oxygen atoms.^24^ U_3_O_8_ has different types of oxygen bonded to uranium, yielding different bond lengths. At room temperature (orthorhombic structure), two uranium atoms [labeled U(1) and U(2)] are surrounded by six oxygen atoms at distances between 2.07 and 2.23 Å, with the seventh oxygen atom bonded to U(1) and U(2) at distances of 2.44 and 2.71 Å, respectively.^25−28^ Loopstra^29^ established that the room-temperature orthorhombic and high-temperature hexagonal structures are similar, except that the hexagonal structure has one uranium site, whereas the orthorhombic structure has two. However, the surrounding oxygens are approximately equivalent for both lattice types.^29^

The crystallographic structure of U_3_O_8_ changes during the heating and cooling processes. There are three phases of U_3_O_8_ having five related polymorphs.^30−34^ There is a general agreement that U_3_O_8_ transforms from an orthorhombic to a hexagonal structure depending on the temperature. Early work (Ackermann et al.^35^) presented detailed data on the U_3_O_8_ lattice parameters and concluded that the stoichiometry of U_3_O_8_ changes continuously, reversibly, and anisotropically above room temperature, with phase transition from an orthorhombic to a hexagonal symmetry at 350 ± 10 °C. On the other hand, later publications (Naito et al.^36^ and Utlak and McMurray^25^) determined that U_3_O_8_ transfers from an orthorhombic to a hexagonal structure in two steps, a first-order transition state from an orthorhombic to a pseudo-hexagonal structure at 295 °C and a second-order transition state at 577 °C to a hexagonal structure. A pseudo-hexagonal structure of U_3_O_8_ at 500 °C was also identified by X-ray diffraction and neutron powder diffraction (Herak^37^).

Our study investigates the oxygen fractionation factor and the extent of the exchange reaction between U_3_O_8_ and atmospheric oxygen in the range of 100–800 °C. The cooling of the samples is performed under vacuum to prevent the retrograde process from altering the oxygen isotopic composition at each temperature. The measured crystallographic changes at this temperature range are discussed in the context of the oxygen fractionation factor and the exchange reactions with atmospheric oxygen, which are involved in the fabrication process of U_3_O_8_.

## 2 Materials and Methods

Three U_3_O_8_ samples with different oxygen isotopic compositions were investigated in this study. Two were synthesized from uranyl nitrate hydrate (UNH); U_3_O_8_-I has a δ^18^O value of 10.02‰ ± 0.31‰, and U_3_O_8_-II has a δ^18^O value of 0.28‰ ± 0.13‰. These two materials were synthesized intentionally differently (at the cooling step) to achieve a difference in their δ^18^O values.^17^ The third sample, U_3_O_8_-III, has a δ^18^O value of 4.78‰ ± 0.50‰, is a natural commercial U_3_O_8_ material purchased from CETAMA, France (commercially known as “CHANTERELLE”), and is used as a calibration standard for impurities.

The samples (100 mg each) were calcined in a tungsten crucible and placed for 4 h in a quartz tube within a tube furnace at a temperature range of 100–800 °C under an atmospheric environment. After 4 h at the desired temperature, vacuum was applied to the quartz tube (∼10^–3^ Torr), and the oven was cooled to room temperature to avoid retrograde exchange reactions with atmospheric oxygen, which can shift the original isotope value.^16,17^

XRD analysis (Rigaku, Ultima III) was performed on samples weighing several milligrams under an atmospheric environment by continuous scanning at 40 kV/40 mA in the range of 10–80° at a rate of 2 °/min. The analyzed samples were those prepared at 100, 300, 400, 500, and 700 °C. In addition, in situ XRD measurements at high temperature were performed on U_3_O_8_-II on a Smart-Lab (Rigaku, Japan) diffractometer equipped with a rotating Cu anode operating at 45 kV and 200 mA with a HyPix-3000 two-dimensional detector in Bragg–Brentano geometry with a variable incident slit. The beam, with a width of 5 mm, was shaped by 2.5° solar before and after the sample measurement. The detector was operated in the 1D mode with scattering and receiving slits equal to 5 mm. The powder was heated in situ with a DHS 1100 heating stage by Anton Paar in an ambient environment. The heating and cooling rates were 5 °/min. The powder was kept at each temperature for 15 min before being measured. Diffraction patterns were measured between 2θ equal to 10–100° with 1.5 °/min.

The DSC (differential scanning calorimeter—DSC823e, Mettler-Toledo) analysis was conducted on U_3_O_8_-III by weighing 4.68 mg into a 100 μL aluminum crucible. The measurement was performed under an atmospheric environment in the temperature range of 30–600 °C at a heating rate of 10 °C/min.

Oxygen isotopic analyses of the U_3_O_8_ samples were conducted using an isotope ratio gas-chromatography-mass spectrometer (irmGCMS, Thermo Scientific Delta Plus Advantage) and an IR CO_2_ laser (10.6 μm, New Wave Research 25 W). The method is described in detail elsewhere.^15,17^ Briefly, U_3_O_8_ samples (1005–1596 μg) and SiO_2_ samples (NBS-28, standard material for quality check and calibration; 260–450 μg) were placed in nickel cups in a stainless steel chamber and heated overnight at 80 °C under high vacuum. Pre-fluorination was performed thrice for the entire cell with 80 Torr of BrF_5_. Samples were reacted by laser heating in 90 Torr of a BrF_5_ atmosphere. The liberated oxygen was purified by liquid nitrogen traps, concentrated on a 5 Å molecular sieve, cooled in liquid nitrogen, and transferred to the mass spectrometer through a gas chromatograph column for isotope measurement in a continuous flow mode. The international SiO_2_ standard NBS-28 (δ^18^O = 9.58‰)^38^ was used for consistency and calibration in each batch. The measured values are expressed in δ-notation in permil, relative to Vienna Standard Mean Ocean Water (VSMOW). The long-term standard deviation (SD) for NBS-28 was 0.30‰. Each U_3_O_8_ sample was run at least in triplicate, and the SD is reported for each sample.

## 3 Results

### 3.1 δ^18^O of U_3_O_8_

The average δ^18^O values of the U_3_O_8_ samples (three to five measurements for each sample), prepared at the temperature range of 100–800 °C, and of the NBS-28 samples are presented in Table 1. The δ^18^O values of the U_3_O_8_ samples as a function of calcination temperatures are plotted in Figure 2. The δ^18^O precision of U_3_O_8_ is identical to the routinely measured NBS-28 standards.

**Figure 1:**
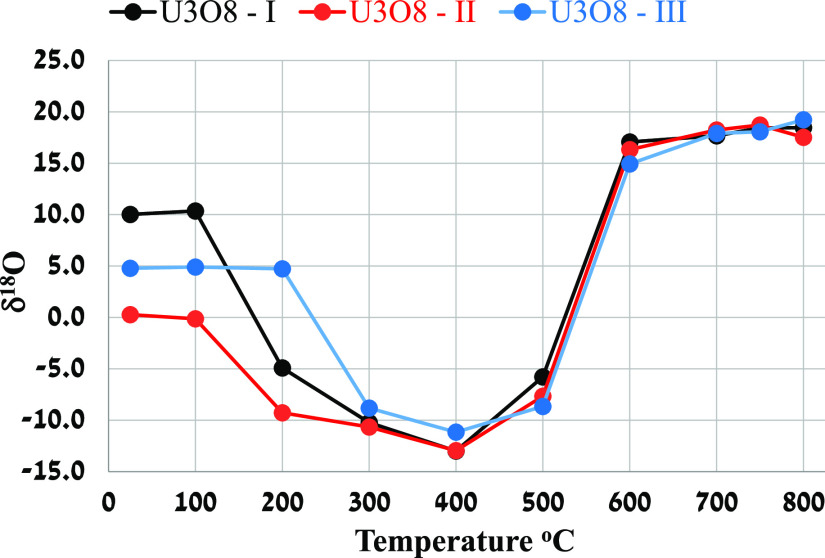
δ^18^O (in ‰ relative to VSMOW) values for the U_3_O_8_ samples at different preparation temperatures.

**Figure 2:**
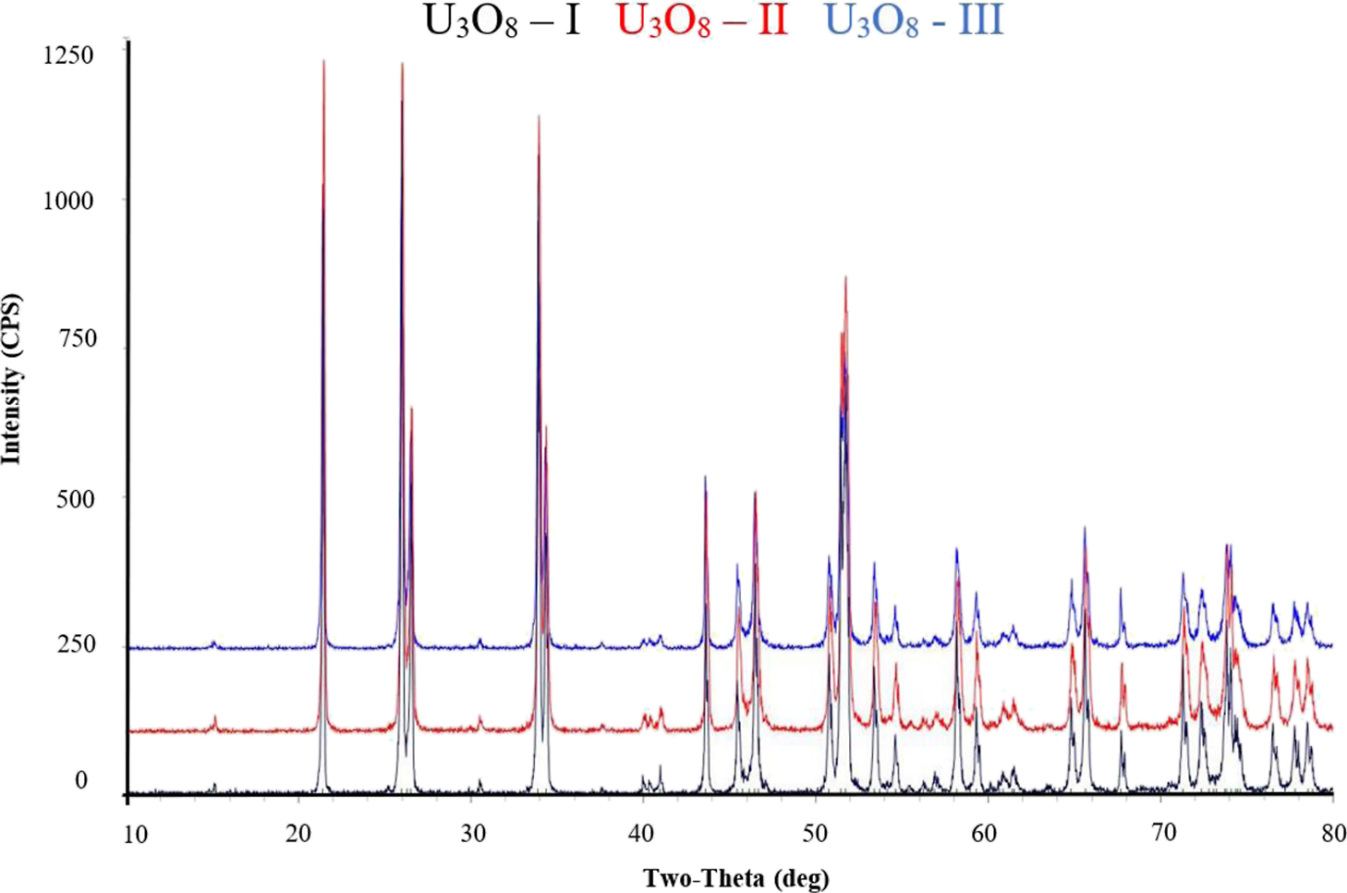
XRD diffractograms of the starting materials U_3_O_8_-I, U_3_O_8_-II, and U_3_O_8_-III at room temperature.

**Table 1:** δ^18^O (in ‰ Relative to VSMOW) Values of the U_3_O_8_ Samples Prepared at Different Temperatures^a^

	U_3_O_8_-I		U_3_O_8_-II		U_3_O_8_-III	
sample temp. (°C)	δ^18^O (‰ VSMOW)	SD (‰)	δ^18^O (‰ VSMOW)	SD (‰)	δ^18^O (‰ VSMOW)	SD (‰)
**25**	**10.02**	**0.31**	**0.28**	**0.13**	**4.78**	**0.50**
100	10.35	0.34	–0.13	0.64	4.90	0.44
200	–4.91	0.57	–9.27	0.81	4.73	0.29
300	–10.23	0.17	–10.66	0.49	–8.82	0.85
400	–13.00	0.35	–12.96	0.36	–11.16	0.33
500	–5.78	0.28	–7.67	0.02	–8.65	0.13
600	17.06	0.19	16.32	0.23	14.93	0.08
700	17.67	0.28	18.24	0.19	17.90	0.38
750	18.43	0.36	18.71	0.36	18.05	0.61
800	18.46	0.59	17.52	0.30	19.23	0.19
NBS-28^b^	9.53‰ ± 0.30‰ (*n* = 21)					

a The “bold” values correspond to the δ^18^O values of the starting U_3_O_8_ samples.

b NBS-28 has an assigned isotope value of 9.58‰ ± 0.09‰ as an international standard.^38^

All samples maintained the original δ^18^O values between 25 and 100 °C, while U_3_O_8_-III maintained the value up to 200 °C. A general isotope depletion trend is evident up to 300–400 °C for all samples. The minimum δ^18^O value occurred around 400 °C. An opposite trend toward heavy δ^18^O values, in the temperature range of 400–800 °C, was measured.

The δ^18^O values obtained at 300 and 400 °C were similar for all the three samples despite the 10‰ difference in the original values. This similarity in δ^18^O values is also maintained in the temperature range of 500–800 °C. The δ^18^O values of the samples prepared at 700 and 800 °C reached an average maximum value of 18.25‰ ± 0.53‰.

### 3.2 Crystal Structure

The XRD diffractograms of the starting U_3_O_8_ samples, U_3_O_8_-I, U_3_O_8_-II, and U_3_O_8_-III, are identical and consist of a single-phase α-U_3_O_8_ (Figure 2).

**Figure 3:**
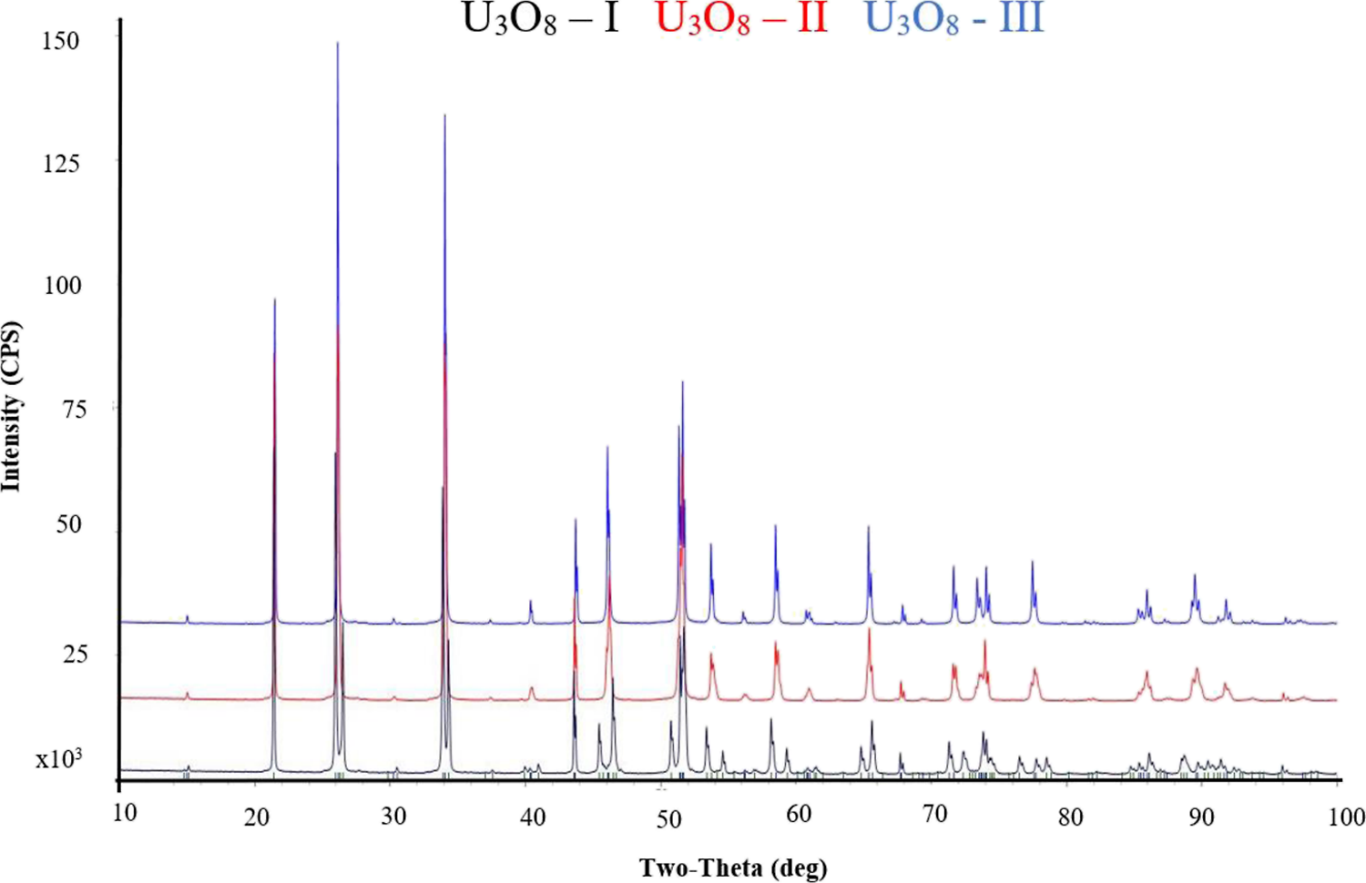
XRD diffractograms of U_3_O_8_-II at temperatures 25, 300, and 750 °C.

**Figure 4:**
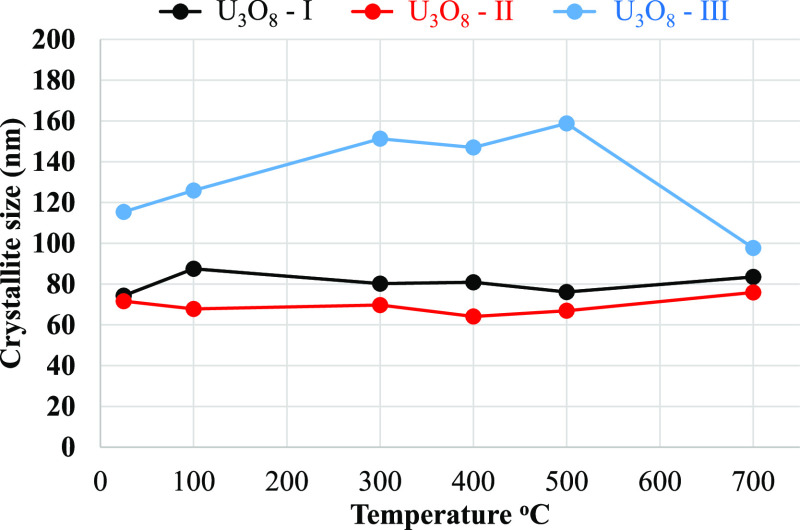
Crystallite size of the U_3_O_8_ starting material. *The estimated error bars are smaller than the markers.

A continuous XRD analysis was performed at the temperature range of 25–800 °C on U_3_O_8_-II. The results (Table 2 and Figure 3) show an orthorhombic structure in the temperature range of 25–200 °C. At 300 °C, two phases, orthorhombic and hexagonal, were obtained. At 400–800 °C, a single hexagonal phase was obtained. The sample was cooled back to room temperature and then remeasured. The cooling back process yielded a single orthorhombic phase. These results present a reversible and continuous structural change, with expansion along the *a*-axis and contraction along the *b* and *c* axes, from the orthorhombic phase to the hexagonal phase at 300 °C. These results agree with the results published by Ackerman et al.^35^ and Miskowiec.^39^ The crystallite sizes of the starting materials were calculated from the XRD measurements (Figure 4). The crystallite size of the synthesized samples, U_3_O_8_-I and U_3_O_8_-II, remains stable and does not change as a function of the preparation temperature. In contrast, the crystallite size of the commercial material, U_3_O_8_-III, increases with the increase of temperature and decreases at 750 °C. The average crystallite sizes of U3O8-I and U3O8-II are 80 and 70 nm, respectively, while U3O8-III has a larger average crystallite size of 132 nm.

**Table 2:** XRD Measurements at Elevated Temperatures of U_3_O_8_-II^a^

temperature °C	phase	*a* Å	*b* Å	*c* Å
25	OR	4.1475 ± 0.0010	11.9601 ± 0.0028	6.7192 ± 0.0016
100	OR	4.1465 ± 0.0009	11.9310 ± 0.0025	6.7331 ± 0.0014
200	OR	4.1474 ± 0.0007	11.8886 ± 0.0018	6.7611 ± 0.0010
300	OR	4.1445 ± 0.0008	11.8373 ± 0.0022	6.7676 ± 0.0013
	H	6.8061 ± 0.0013	6.8061 ± 0.0013	4.1428 ± 0.0008
400	H	6.8134 ± 0.0007	6.8134 ± 0.0007	4.1436 ± 0.0004
500	H	6.8174 ± 0.0007	6.8174 ± 0.0007	4.1429 ± 0.0004
600	H	6.8201 ± 0.0007	6.8201 ± 0.0007	4.1415 ± 0.0005
700	H	6.8233 ± 0.0007	6.8233 ± 0.0007	4.1405 ± 0.0004
800	H	6.8274 ± 0.0007	6.8273 ± 0.0007	4.1403 ± 0.0004
back to 25	OR	4.1457 ± 0.0008	11.9544 ± 0.0023	6.7163 ± 0.0013

a H—hexagonal and OR—orthorhombic.

**Figure 5:**
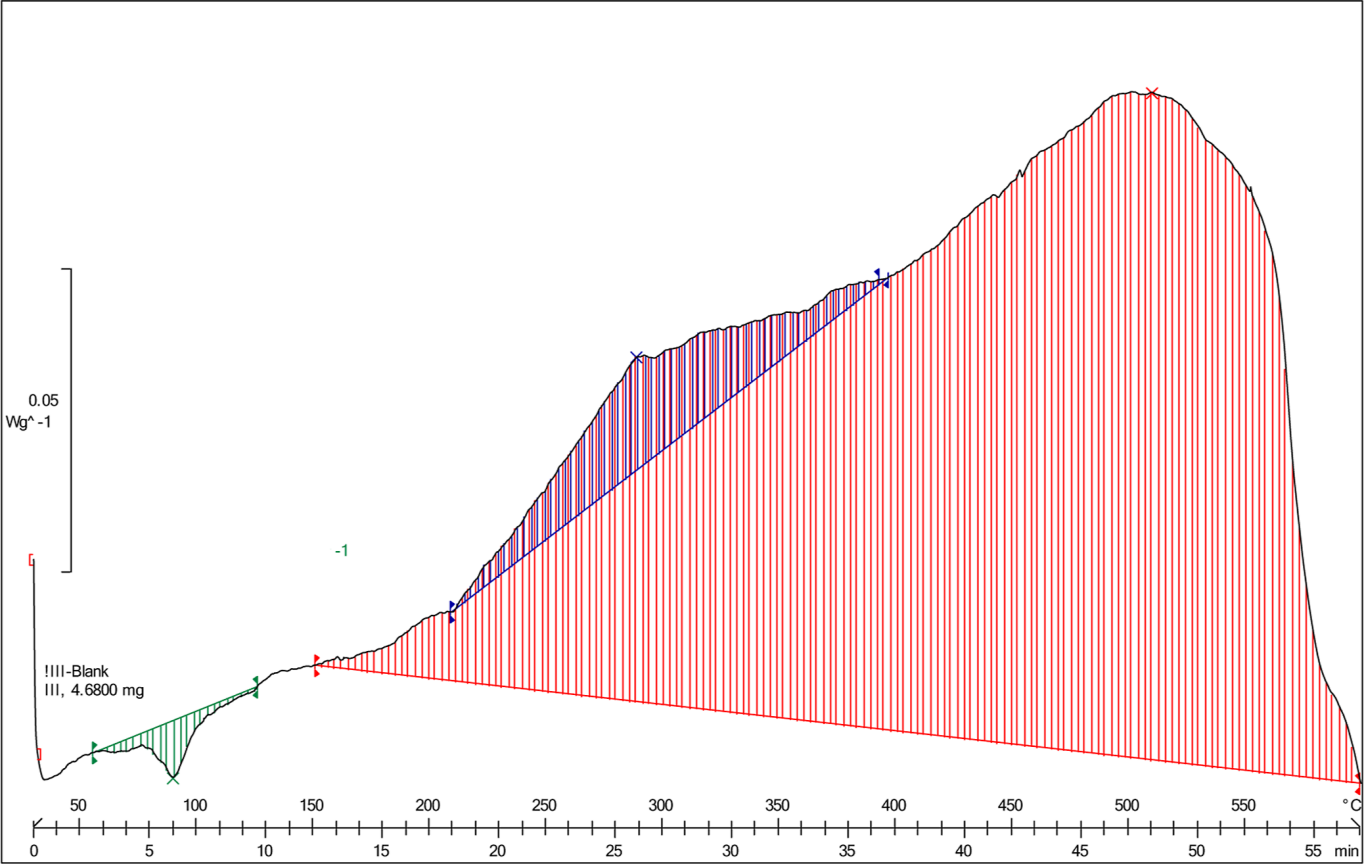
DSC analysis of U_3_O_8_-III at temperatures 30–600 °C. The colors are explained in the text.

DSC analysis was performed on U_3_O_8_-III. The results (Figure 5) exhibit two exothermal peaks at 289 and 510 °C associated with heat capacities of 10.16 J g^–1^ (8.56 J mol^–1^, the blue area) and 163.08 J g^–1^ (137.13 J mol^–1^, the red area), respectively. The second peak, at 510 °C, indicates second-order phase transitions. These results are in good agreement with the published literature.^36^ However, Hideaki et al.^36^ reported a heat capacity of 148 J mol^–1^ at 295 °C and 314 J mol^–1^, which might result from the heat capacity peak dependence on the heating rate.

## 4 Discussion

The key observations following the experiments which were conducted are as follows: (1) the non-monotonic change in δ^18^O with increasing temperature; (2) the similarity among the δ^18^O values from 300 °C to 800 °C, diminishing the original 10‰ difference among the samples; and (3) the occurrence of phase transition during the heating process. Thus, it is essential to evaluate whether the isotope equilibrium between U_3_O_8_ and atmospheric oxygen was attained and, as a consequence, whether a fractionation factor at each temperature can be calculated.

### Fractionation Factor and Percent of Exchange

In order to determine the fractionation factor between U_3_O_8_ and atmospheric oxygen as well as its dependency on the temperature, we adopted the method developed by Northrop and Clayton.^40^ It is based on the oxygen isotope exchange between two substances, considering the forward and reverse reaction rates and the isotope mass balance considerations. Equation 1 describes the partial exchange between a solid and a gas phase and allows the determination of whether the isotope equilibrium was attained or its proximity to the isotope equilibrium fractionation for a set of companion exchange runs (minimum of three samples), differing only by their initial δ^18^O.
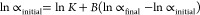
1where α is the oxygen isotopic fractionation between U_3_O_8_ and atmospheric O_2_ and *K* is the equilibrium fractionation of the system.

Hence, a plot of (ln ∝_final_ – ln ∝_initial_) against (ln ∝_initial_) will give a straight line of slope *B* and an intercept of ln *K*. If equilibrium is attained in all samples of a set, ln ∝_final_ = ln *K* and *B* = −1. For samples that are not yet at the isotope equilibrium, *B* will lie between −1 and infinity. In addition, the percent of exchange can be calculated from the slope by (−100/*B*).

A set of three studied companion samples are shown to approach equilibrium from opposite directions at each temperature, satisfying the conditions required to apply this approach. The calculations (Table 3) are based on the data provided in Table 1 and a δ^18^O value of an atmospheric oxygen of 23.5‰.^41^

**Table 3:** Fractionation Factor and Percent of Exchange for the System of U_3_O_8_ and Atmospheric Oxygen at Different Temperatures

temp. °C	100	200	300	400	500	600	700	750	800
ln *K*	–0.018	–0.020	–0.033	–0.035	–0.033	–0.007	–0.006	–0.005	–0.004
*K*	0.982	0.980	0.967	0.966	0.968	0.993	0.994	0.995	0.996
*B*	12.290 ± 3.600	–0.253 ± 0.593	–0.996 ± 0.203	–0.943 ± 0.203	–1.167 ± 0.327	–1.040 ± 0.231	–0.946 ± 0.007	–0.971 ± 0.058	–1.069 ± 0.174
% exchange			100.4	106.1	85.7	96.1	105.8	102.9	93.5
1000 ln α_U_3_O_8_-atm. oxygen_			–33.13	–34.68	–32.70	–6.81	–6.15	–5.37	–4.08

The values of *B* (Table 3) for all temperatures indicate that the isotope equilibrium in the U_3_O_8_–atmospheric oxygen system was attained for the samples exposed to temperatures of 300–800 °C. The calculated *B* values are very close to −1, as expected from the theoretical isotope formulation.^40^ The calculated percent of exchange is also around 100%, corresponding to the *B* values. This conclusion coincides with the observation (Figure 1) that the initial δ^18^O value was diminished at these temperatures, and similar values for all the three samples were obtained. In contrast, the *B* values for 100 and 200 °C are far from −1, indicating that the system did not reach the isotope equilibrium and, therefore, the calculation of 1000 ln α is invalid. The similarity between the δ^18^O values of U_3_O_8_ obtained at 25 °C to the values measured at 100 °C suggests that no exchange reaction with the atmospheric oxygen occurred. At 200 °C, samples U_3_O_8_-I and U_3_O_8_-II exhibit a similar trend toward lighter δ^18^O values, while the δ^18^O value of U_3_O_8_-III remains stable (4.73‰ ± 0.29‰) concerning the starting value. These results suggest a partial exchange reaction with the atmospheric oxygen at 200 °C, which probably originates from the crystallite size and/or surface area, two parameters that affect exchangeability. Applying the *B* criteria to 100 and 200 °C implies that the system at these temperatures has not reached the isotope equilibrium; therefore, the fractionation factor, 1000 ln α, and the percent of exchange are not calculated for these temperatures.

**Figure 6:**
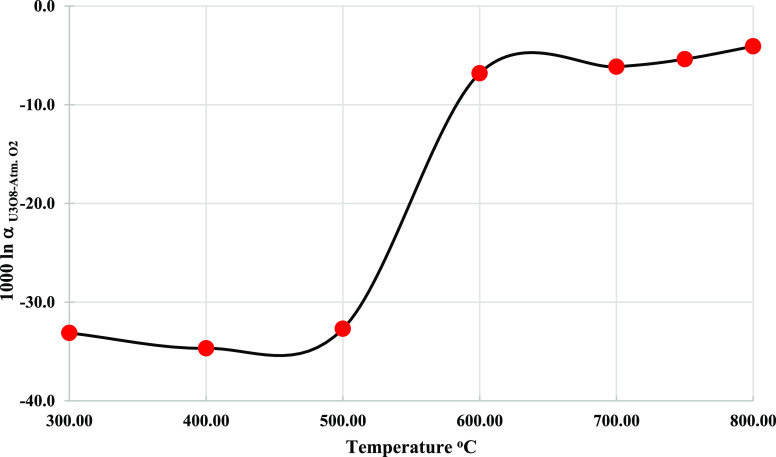
Fractionation factor of U_3_O_8_ and atmospheric oxygen in the temperature range of 300–800 °C. *The estimated error bars are smaller than the markers.

The calculated fractionation factors (1000 ln α_U_3_O_8_-atm. O_2__) in the temperature range of 300–800 °C (Table 3 and Figure 6) show two distinct groups: the first at the temperature range of 300–500 °C with values between −34.68 and −32.70‰ and the second at the temperature range of 600–800 °C with values between −6.81 and −4.08‰. A sharp transition between these groups occurs between 500 and 600 °C.

Such unique trends in the δ^18^O values of the U_3_O_8_ samples were observed only in a few minerals,^24^ suggesting that the structural change in the lattice is the main cause of that behavior.

The sharp change in the equilibrium fractionation factor coincides with the phase transition from the orthorhombic to hexagonal structure, which occurs at 500–577 °C.^25,36,37^ The XRD measurements showed a transition state from the orthorhombic to hexagonal phase at 300–400 °C, close to the values reported by Ackermann et al.,^35^ Girdhar and Westrum,^42^ and Notz, Huntington, and Burkhardt.^43^ Apparently, some XRD measurements fail to identify the second-order transition state, which is related to the presence of a pseudo-hexagonal structure that evolves from the orthorhombic structure.^28^

In contrast to XRD, DSC measurements identified the two transition states at 295 and 510 °C, which are related to the two transitions from orthorhombic to pseudo-hexagonal to hexagonal. Thus, we attribute the first-order transition state, at 300–400 °C, to the fact that the δ^18^O values reach a minimum and the second-order transition state, at 500–600 °C, to the inverse trend of the oxygen isotopic composition. We consider the possible isotope exchange with humidity H_2_O at 400 °C (Klosterman et al.^22^) minor to the isotope effect of the observed phase transition.

The temperature dependency of the fractionation factor is not common and does not resemble the published curves of most minerals of geological interest. The increase of 1000 ln α with increasing temperature up to 400 °C, despite the full exchange for the orthorhombic phase, is unusual. This might be explained by the uranium mass effect, which is the tendency of heavy atoms (uranium) to favor the light isotope in order to lower the total free energy of the system by reducing the vibrational frequencies of the bonds.^24,44,45^ Above 400 °C, the fractionation factor is minimized with increasing temperature, and the δ^18^O (18.4‰) value of U_3_O_8_ reaches the isotope value of atmospheric O_2_ (23.5‰). It is possible that the uranium mass effect causes the slight isotope depletion at high temperatures. The decrease in the percent of exchange at 800 °C may result from the loss of oxygen occurring above 750 °C.

## 5 Conclusions

The isotope fractionation between U_3_O_8_ and atmospheric O_2_ was quantified by removing the retrograde effect during the cooling stage at the end of the fabrication process of U_3_O_8_ from uranyl nitrate hydrate or heating U_3_O_8_ under atmospheric conditions. We find that the U_3_O_8_–atmospheric oxygen system attains the isotope equilibrium at 300 °C and maintained the isotope equilibrium conditions up to 750–800 °C, where U_3_O_8_ starts to lose oxygen. The temperature dependency of the fractionation factor exhibits two distinct regions which correspond to the structural changes that U_3_O_8_ undergoes at the temperature range of 25–800 °C. The minimum of δ^18^O values which were obtained at 400 °C is associated with the pseudo-hexagonal structure, and the second step, in the temperature range of 500–800 °C, is a clear trend toward heavier δ^18^O which can be related to the hexagonal structure. The significant shift in the oxygen isotopic fractionation (α), in the temperature range of 500–600 °C, coincides with the second-order transition state from the pseudo-hexagonal to the hexagonal structure. The fact that the δ^18^O value of U_3_O_8_ does not reach the isotope value of atmospheric oxygen at the very high temperatures might be attributed to the uranium mass effect.
